# A Dual-Channel Architecture Based on GCN and HGCN for Dynamic Link Prediction

**DOI:** 10.3390/e28080912

**Published:** 2026-08-14

**Authors:** Bing Wu, Sheng Zhang, Jiangnan Zhou, Mengen Xu, Qiuming Wang, Yirong Zeng, Ka Sun, Fenglian Yuan

**Affiliations:** School of Information Engineering, Nanchang Hangkong University, Nanchang 330063, China; wb020112@163.com (B.W.); 2404085404312@stu.nchu.edu.cn (J.Z.); 2404085404305@stu.nchu.edu.cn (M.X.); 2404085404315@stu.nchu.edu.cn (Q.W.); 2504081200002@stu.nchu.edu.cn (Y.Z.); 70308@nchu.edu.cn (K.S.); yuanfenglian@nchu.edu.cn (F.Y.)

**Keywords:** dynamic networks, dual-channel, graph convolutional networks, hypergraph convolutional networks, link prediction

## Abstract

Dynamic link prediction, which aims to infer future edges from historical network structures, is a fundamental task in dynamic network analysis. Traditional models fail to capture high-order information, while existing methods neglect the distinct temporal evolution patterns between low-order and high-order structures, thereby limiting prediction accuracy. To address these issues, we propose DC-GHCN, a dynamic link prediction model based on a dual-channel architecture that integrates Graph Convolutional Network (GCN) and Hypergraph Convolutional Network (HGCN). Firstly, we extract closed motifs from dynamic network snapshots to construct an initial hypergraph, then refine it via nested motif pruning and node weight compensation strategies. Secondly, we design a dual-channel architecture: the GCN channel learns low-order structural features, while the HGCN channel learns high-order structural features. Furthermore, two independent Gated Recurrent Units (GRUs) separately model the temporal evolution of the two channels. Finally, the model employs a gating mechanism to adaptively fuse the dual-channel node representations for link prediction. Experiments on five real-world dynamic network datasets demonstrate that DC-GHCN outperforms baseline models, validating the effectiveness of the proposed model in dynamic link prediction.

## 1. Introduction

Numerous real-world complex systems, such as social networks, biological metabolic networks, and financial transaction flows, can be naturally modeled as complex networks [1], where nodes and edges represent the entities in the system and their intricate interactions, respectively. In practice, these networks are rarely static; instead, they evolve continuously with dynamic topological structures and temporal patterns [2]. To accurately characterize such systems, researchers typically define dynamic networks as a sequence of discrete time snapshots. Within the research paradigm of dynamic networks, link prediction aims to infer edges that emerge at future time steps by leveraging the evolutionary patterns of historical network topologies [3]. As a core task in network science and data mining, dynamic link prediction serves as a critical enabling technology for a wide range of real-world applications, including social friend recommendation [4], dynamic regulatory network analysis of biological proteins [5], and anomalous financial transaction and money laundering detection [6].

Early link prediction studies were predominantly based on heuristic node similarity models. Such models estimate the likelihood of edge formation by measuring specific attributes of the shared neighborhood between two target nodes. Typical representatives include Common Neighbors (CN), Adamic-Adar (AA) [7], and the Jaccard Coefficient (JC) [8]. With the advancement of deep learning and graph embedding techniques, Graph Neural Networks (GNNs) have gradually become the mainstream paradigm for link prediction. Representative architectures such as Graph Convolutional Networks (GCNs) [9] and Graph Attention Networks (GATs) [10] achieve substantial performance gains in link prediction by aggregating local neighborhood information. Nevertheless, these models are designed for static networks and cannot directly capture temporal patterns in dynamic networks. To capture the spatio-temporal evolution of dynamic networks, researchers have explored the deep integration of spatial GNNs with temporal sequence modeling methods [11,12]. One classic strategy is to combine Recurrent Neural Networks (RNNs) and their variants. For instance, Chen et al. [13] proposed the GC-LSTM model, which embeds graph convolution operations into the internal update mechanism of Long Short-Term Memory (LSTM) networks. It captures spatio-temporal dynamic features by jointly recording the historical hidden states of nodes and their neighbors. To improve model robustness against frequent topological changes and unseen nodes, Pareja et al. [14] proposed the EvolveGCN model. Departing from the conventional paradigm of directly evolving node embedding vectors, this model innovatively uses RNNs to evolve the parameter weight matrices of GCN over time, resulting in two variants: EvolveGCN-O (focused on parameter updates) and EvolveGCN-H (incorporating hidden states). Following the remarkable success of self-attention mechanisms in sequence modeling, dynamic GNNs based on the Transformer architecture have emerged. Sankar et al. [15] proposed the DySAT model, which abandons the traditional recursive RNN structure. By alternately stacking structural and temporal self-attention layers, it effectively captures the local spatial structure within individual time snapshots and computes node evolution patterns across multiple snapshots in parallel, significantly enhancing the model’s ability to model long-range temporal dependencies. As research on network topology mining deepens, cutting-edge studies have started to focus on high-order topological structures in networks. Wu et al. [16] proposed the TMAGCN model, which addresses the limitation of traditional models that rely exclusively on pairwise binary edge interactions. By integrating high-order motifs into node representations and performing joint spatio-temporal modeling, the work demonstrates the critical role of high-order topology in improving link prediction performance. Recent research has also explored the integration of heterogeneous data and domain knowledge. For example, Song et al. [17] proposed a generative data–physics fusion framework for hierarchical reliability assessment under limited data, which incorporates physical consistency constraints into generative models to characterize multi-site responses and interdependent failure modes.

However, existing models still suffer from two critical limitations. First, the unfiltered incorporation of high-order motifs inevitably introduces structural redundancy. In locally dense topological regions in particular, nested structures are pervasive among motifs of different orders. Treating all such motifs as independent structural units for information aggregation not only results in information redundancy but also incurs unnecessary computational overhead. Second, low-order and high-order structures in dynamic networks present disparate temporal evolutionary patterns: low-order pairwise edges generally evolve at a faster pace, while high-order closed topological structures are more temporally stable. Coupled modeling of these two types of information—as seen in TMAGCN and other dual-stream dynamic graph models—typically employs an early-fusion paradigm where low-order and high-order representations share a common temporal parameter space. This shared-parameter recurrent modeling is technically suboptimal: the high-frequency volatility of low-order pairwise edges often dominates the gradient updates during training, inadvertently washing out the temporally stable, low-frequency topological invariants of high-order closed motifs. Consequently, such coupled architectures suffer from parameter interference and fail to disentangle their disparate evolutionary dynamics.

To address the aforementioned issues, we propose a novel dynamic link prediction model named DC-GHCN. The model first constructs an initial hypergraph by extracting closed motifs from raw network snapshots. To mitigate structural redundancy arising from nested motifs, it prunes 3-order motifs subsumed by 4-order motifs and introduces a node weight compensation strategy to encode the information of pruned motifs into retained hyperedges. This structural refinement reduces redundant feature propagation while preserving essential local topological information. Subsequently, the model constructs a dual-channel encoding architecture: the GCN channel learns low-order structural features from original snapshots, while the HGCN channel extracts high-order structural features from the refined hypergraphs. Technically distinct from existing dual-stream models, DC-GHCN explicitly mitigates the aforementioned parameter interference through a decoupled, late-fusion temporal architecture. Each channel is equipped with an independent GRU, maintaining strictly isolated parameter spaces for low-order and high-order temporal modeling. This design guarantees that the fast-evolving low-order structural gradients do not interfere with the stable high-order topological features during temporal backpropagation. Finally, a gating mechanism adaptively fuses the dual-channel representations into comprehensive node embeddings, which are fed into a multi-layer perceptron (MLP) to fulfill link prediction at the next time step.

The main contributions of this paper are summarized as follows:

(1) We propose a novel hypergraph construction method that enhances the model’s capability to extract high-order structural information. In this method, the hypergraph is constructed using closed motifs and further refined through nested motif pruning and node weight compensation.
(2) We design a link prediction model, which utilizes a GCN to capture low-order structural features, employs an HGCN to extract high-order structural features, and uses independent GRUs to model their temporal evolution separately. Through a gating mechanism, it adaptively fuses these features, yielding more comprehensive node representations to enhance link prediction accuracy.

The rest of this paper is organized as follows: Section 2 provides the relevant definitions and problem description, including key concepts such as dynamic networks, motifs, and hypergraphs, laying a theoretical basis for the proposed model. Section 3 details the overall framework and specific implementation of the DC-GHCN model, elaborating on three core modules: hypergraph construction, dual-channel encoding, and adaptive fusion. Section 4 presents the experimental evaluations, including dataset descriptions, baseline model configurations, and experimental setups, followed by an in-depth analysis of the overall results, parameter sensitivity, ablation studies, and comparisons of hypergraph construction strategies. Finally, Section 5 concludes the paper, highlighting the contributions of the proposed model and outlining future research directions.

## 2. Preliminaries

### 2.1. Related Definition

#### 2.1.1. Dynamic Network

A dynamic network, denoted as G, can be formalized as a sequence of chronologically ordered network snapshots $G=\{G^{\left(1\right)},G^{\left(2\right)},\ldots ,G^{\left(T\right)}\}$, where T represents the total number of time steps. For any time step t∈[1,2,…,T], the corresponding network snapshot is defined as $G^{\left(t\right)}=(V,E^{\left(t\right)})$, where $V=\{v_{1},v_{2},\ldots ,v_{N}\}$ is the set of nodes, $E^{\left(t\right)}\subseteq V\times V$ constitutes the set of edges at time step t. The structural information of each snapshot $G^{\left(t\right)}$ is represented by its adjacency matrix $A^{\left(t\right)}\in R^{N\times N}$, where entry $A_{ij}^{\left(t\right)}=1$ if node $v_{i}$ and $v_{j}$ are connected at time t, and $A_{ij}^{\left(t\right)}=0$ otherwise.

#### 2.1.2. Motif

**Figure 1:**
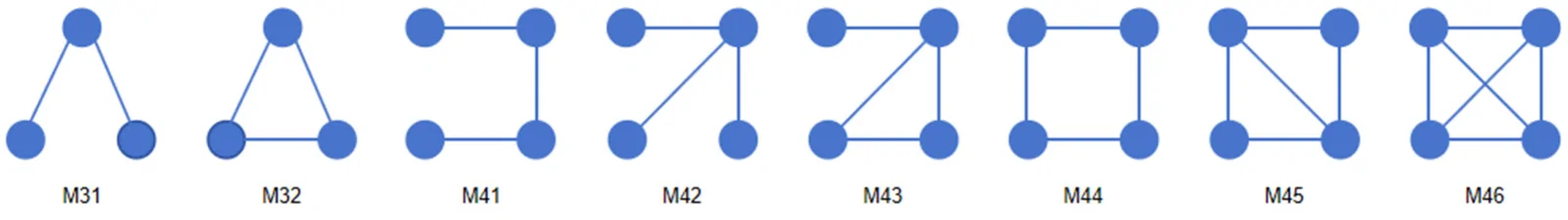
All 3-order and 4-order motifs in the network.

Motifs [18] are defined as small-scale induced subgraphs that occur significantly and frequently in complex networks, capable of characterizing specific local topological patterns. Related studies [19] have demonstrated that nodes within closed motifs typically exhibit higher similarity and stronger structural cohesion, enabling them to accurately characterize dense local community features in real-world networks. Regarding the selection of specific orders, we extract 3-order and 4-order closed motifs, which correspond to M32, M44, M45, and M46 in Figure 1. 3-order and 4-order motifs can effectively capture high-order interaction patterns. Conversely, for higher-order motifs of the 5-order and beyond, the computational overhead for their extraction increases exponentially with the motif order. Therefore, selecting 3-order and 4-order closed motifs ensures the capture of core high-order features while controlling the computational complexity of the algorithm.

#### 2.1.3. Hypergraph

To explicitly represent the multi-node joint relationships corresponding to motifs, we adopt hypergraph theory [20,21]. Unlike simple graphs where an edge can only connect two nodes, a hyperedge in a hypergraph can connect multiple nodes simultaneously. This property makes it a natural tool for characterizing local high-order group interactions.

A hypergraph can be defined as H=(V,E), where $V=\{v_{1},v_{2},\ldots ,v_{N}\}$ denotes the vertex set composed of N entities, and $E=\{\varepsilon_{1},\varepsilon_{2},\ldots ,\varepsilon_{M}\}$ represents the set consisting of M hyperedges. Distinct from traditional graph structures, any hyperedge $\varepsilon_{j}\in E$ in a hypergraph is a subset of the vertex set V, capable of connecting two or more vertices simultaneously.

- Incidence Matrix

The topological structure of a hypergraph H is described by an incidence matrix $B\in R^{N\times M}$, where N and M denote the number of vertices and hyperedges, respectively. The element B(i,j) in the matrix is defined as follows:

(1) $$B(i,j)=\left\{\begin{array}{c} 1,\mathrm{if}v_{i}\in \varepsilon_{j}\\ 0,\mathrm{otherwise} \end{array}\right.$$

- Vertex Degree Matrix

The vertex degree matrix $D_{v}\in R^{N\times N}$ is a diagonal matrix where each diagonal element represents the number of hyperedges connected to a specific vertex. Based on the incidence matrix B defined in Equation (2), it is calculated as follows:

(2) $$D_{v}(i,i)=\sum_{j=1}^{M}B(i,j)$$

- Hyperedge Degree Matrix

The hyperedge degree matrix $D_{\varepsilon }\in R^{M\times M}$ is a diagonal matrix where each diagonal element denotes the number of vertices contained within a specific hyperedge. Derived from Equation (3), its entries are computed as:

(3) $$D_{\varepsilon }(j,j)=\sum_{i=1}^{N}B(i,j)$$

### 2.2. Problem Description

Dynamic network link prediction aims to infer future edges by exploiting the topological evolution patterns of historical networks. Formally, given a sequence of historical snapshots $\left\{G^{\left(1\right)},G^{\left(2\right)},\ldots ,G^{\left(t\right)}\right\}$ and their corresponding adjacency matrices ${\{A}^{\left(1\right)},A^{\left(2\right)},\ldots ,A^{\left(t\right)}\}$ up to time step t, the task goal is to learn a mapping function f(⋅) that predicts the network topology at the next time step:

(4) $${\hat{A}}^{\left(t+1\right)}=f(A^{\left(1\right)},A^{\left(2\right)},\ldots ,A^{\left(t\right)})$$

where ${\hat{A}}^{\left(t+1\right)}\in R^{N\times N}$ is the model-predicted probability matrix, with each entry ${\hat{A}}_{ij}^{\left(t+1\right)}\in \left[0,1\right]$ indicating the likelihood of an edge forming between nodes $v_{I}$ and $v_{j}$ at time step t+1.

## 3. Model

### 3.1. Overall Framework

**Figure 2:**
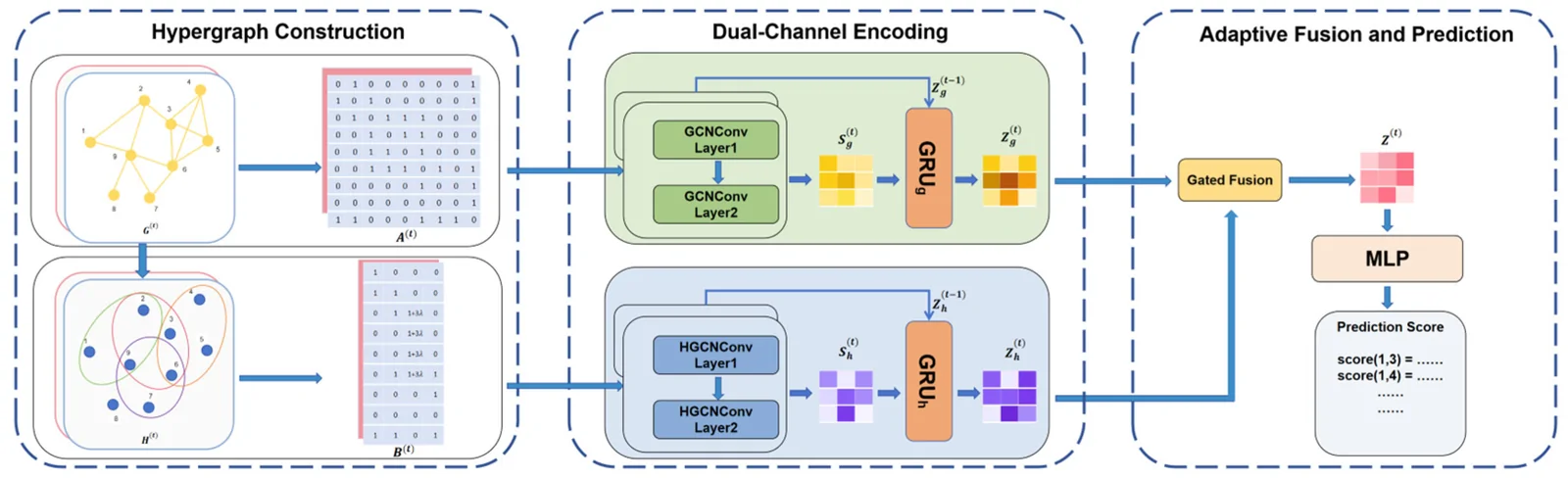
The overall framework of the DC-GHCN model.

This section introduces the framework of the DC-GHCN model. As illustrated in Figure 2, the model fundamentally consists of three core modules: hypergraph construction module, dual-channel encoding module, and adaptive fusion module.

Specifically, given a sequence of the previous t historical network snapshots, the hypergraph construction module first extracts 3-order and 4-order closed motifs at each time step to construct the initial hypergraph. It then implements nested motif pruning to mitigate topological overlap and employs node weight compensation strategy to offset the loss of local information, thereby generating the refined weighted incidence matrix. In the dual-channel encoding module, the GCN channel captures low-order structural features and models their temporal evolution via a dedicated GRU, generating low-order spatio-temporal representations. Meanwhile, the parallel HGCN channel captures high-order structural features, modeling their temporal evolution through an independent GRU. Finally, the adaptive fusion module utilizes a gating mechanism to calculate adaptive weights for fusing the dual-channel node representations, and employs an MLP to score candidate node pairs, ultimately completing the link prediction task.

### 3.2. Hypergraph Construction

#### 3.2.1. Motif Extraction and Hyperedge Mapping

Closed motifs are defined as subgraphs in which every node has a degree of at least two within the subgraph, thereby forming closed structures such as cycles, cliques, or near-cliques without any pendant nodes. As closed motifs constitute densely connected local topological substructures with high internal cohesion in networks, leveraging them as building blocks for hypergraph construction not only enables accurate capture of high-order topological features but also prevents the introduction of sparse, uninformative subgraphs, thereby constraining the overall time complexity of the algorithm. Therefore, we focus on two categories of local high-order patterns in complex networks: 3-order and 4-order closed motifs (corresponding to M32, M44, M45, and M46 in Figure 1).

Specifically, for any network snapshot $G^{\left(t\right)}$ at time step t, we first extract all 3-order and 4-order closed motifs from the topology, denoted as $M_{3}^{\left(t\right)}$ and $M_{4}^{\left(t\right)}$ respectively. The full motif set is given by:

(5) $$M^{\left(t\right)}=M_{3}^{\left(t\right)}\cup M_{4}^{\left(t\right)}$$

For each motif $m_{k}^{\left(t\right)}\in M^{\left(t\right)}$, let $V(m_{k}^{\left(t\right)})$ be the set of vertices covered by the motif. We map each motif to a corresponding hyperedge $\varepsilon_{k}^{\left(t\right)}=V(m_{k}^{\left(t\right)})$, yielding the initial hyperedge set:

(6) $$E_{init}^{\left(t\right)}=\{\varepsilon_{k}^{\left(t\right)}|m_{k}^{\left(t\right)}\in M^{\left(t\right)}\}$$

The resulting initial hypergraph is $H_{init}^{\left(t\right)}=(V,E_{init}^{\left(t\right)})$, with its initial incidence matrix $B_{init}^{\left(t\right)}$ defined as:

(7) $$B_{init}^{\left(t\right)}(i,k)=\left\{\begin{array}{c} 1,\;\mathrm{if}\;v_{i}\in \varepsilon_{k}^{\left(t\right)},\\ 0,\;\mathrm{if}v_{i}\notin \varepsilon_{k}^{\left(t\right)}. \end{array}\right.$$

This mapping transforms closed motifs from original snapshots into hyperedges, which encode high-order topological interactions among multiple nodes.

#### 3.2.2. Nested Motif Pruning

Although the hyperedge mapping process in Section 3.2.1 has transformed all closed motifs into hyperedges within the hypergraph, this unfiltered construction ignores the nested relationships among topological structures. In complex networks, topological nesting phenomenon frequently exists among motifs of different orders. For instance, the 4-order closed motifs illustrated in Figure 1 (e.g., M46) naturally subsume multiple 3-order closed motifs (e.g., M32). If all these motifs are mapped into hyperedges, the subsequent hypergraph convolutions will repeatedly aggregate features from overlapping local regions, resulting in information redundancy.

To address this issue, we propose a nested motif pruning strategy: removing 3-order motifs that are subsumed by 4-order motifs. Formally, given a 3-order motif $\tau \in M_{3}^{\left(t\right)}$ and a 4-order motif $\phi \in M_{4}^{\left(t\right)}$, τ is identified as a nested motif and pruned if V(τ)⊂V(ϕ). The set of all pruned 3-order motifs is defined as:

(8) $$M_{3,drop}^{\left(t\right)}=\{\tau \in M_{3}^{\left(t\right)}|\exists \phi \in M_{4}^{\left(t\right)},V(\tau )\subset V(\phi )\}$$

Correspondingly, the set of retained 3-order motifs is $M_{3,keep}^{\left(t\right)}=M_{3}^{\left(t\right)}\setminus M_{3,drop}^{\left(t\right)}$, and the final retained motif set is given by:

(9) $$M^{*(t)}=M_{4}^{\left(t\right)}\cup M_{3,keep}^{\left(t\right)}$$

We thus obtain the refined hypergraph $H^{\left(t\right)}$, whose hyperedge set $E^{\left(t\right)}$ is given by:

(10) $$E^{\left(t\right)}=\{V(m)|m\in M^{*(t)}\}$$

#### 3.2.3. Node Weight Compensation

While pruning nested 3-order motifs mitigates structural redundancy, it inevitably incurs partial loss of local topological information, such as the connection strength of nodes. To compensate for this loss, we propose a node weight compensation strategy, which incorporates the information of pruned 3-order motifs into the corresponding nodes in retained hyperedges by adjusting the incidence matrix weights.

For a retained hyperedge $\varepsilon_{k}^{\left(t\right)}$ and a node $v_{i}\in \varepsilon_{k}^{\left(t\right)}$, the compensation count $c^{\left(t\right)}(i,k)$ is defined as the number of pruned 3-order motifs that contain $v_{i}$ and whose vertex sets are strict subsets of $\varepsilon_{k}^{\left(t\right)}$:

(11) $$c^{\left(t\right)}(i,k)=\left|\left\{\tau \in M_{3,drop}^{\left(t\right)}|v_{i}\in V(\tau ),V(\tau )\subset \varepsilon_{k}^{\left(t\right)}\right\}\right|$$

A pruned 3-order motif may be simultaneously contained in multiple retained 4-order motifs. In this case, we adopt a non-exclusive compensation strategy rather than arbitrarily assigning the motif to only one hyperedge. According to Equation (11), the pruned motif contributes to the compensation count $c^{\left(t\right)}(i,k)$ of every retained hyperedge $\varepsilon_{k}^{\left(t\right)}$ that contains it, and the resulting count is then scaled by λ in Equation (12). Therefore, no additional tie-breaking rule is required. Since a triangle shared by multiple 4-order motifs participates in several overlapping closed structures, retaining its contribution in all associated hyperedges helps preserve the corresponding local structural overlap and cohesion after pruning. On this basis, the entries of the final refined weighted incidence matrix $B^{\left(t\right)}$ are given by:

(12) $$B^{\left(t\right)}(i,k)=\left\{\begin{array}{ll} 1+\lambda c^{\left(t\right)}(i,k), & if\;v_{i}\in e_{k}^{\left(t\right)},\\ 0, & otherwise. \end{array}\right.$$

where λ∈[0,1] is the compensation coefficient. Specifically, λ=0 corresponds to the case where only nested motif pruning is executed without any weight compensation.

The linear compensation form in Equation (12) is designed as a simple and interpretable additive approximation of the local structural information removed by motif pruning. The baseline value of 1 preserves the original node-hyperedge membership, while each pruned 3-order motif involving node $v_{i}$ within hyperedge $\varepsilon_{k}^{\left(t\right)}$ represents one additional unit of lost local closed-structure information. Therefore, $c^{\left(t\right)}(i,k)$ measures the amount of locally removed structural evidence, and λ controls the contribution of each unit to feature propagation. Compared with a nonlinear transformation, this linear form preserves monotonicity and introduces no additional parameters or functional assumptions.

Importantly, the compensation count is defined locally for each node–hyperedge incidence rather than accumulated from the total number of motifs in the network. For a retained 4-order hyperedge, a given node can belong to at most three distinct 3-order subsets. Therefore, $c^{\left(t\right)}(i,k)$ is bounded by 3, and, with λ∈[0,1], each nonzero incidence weight remains between 1 and 4. Consequently, a large global motif count does not lead to unbounded incidence weights. The compensation mechanism intentionally assigns moderately stronger propagation weights to overlapping dense structures in order to recover information lost during pruning; however, this structural bias is controlled by both the local upper bound of $c^{\left(t\right)}(i,k)$ and the coefficient λ.

**Figure 3:**
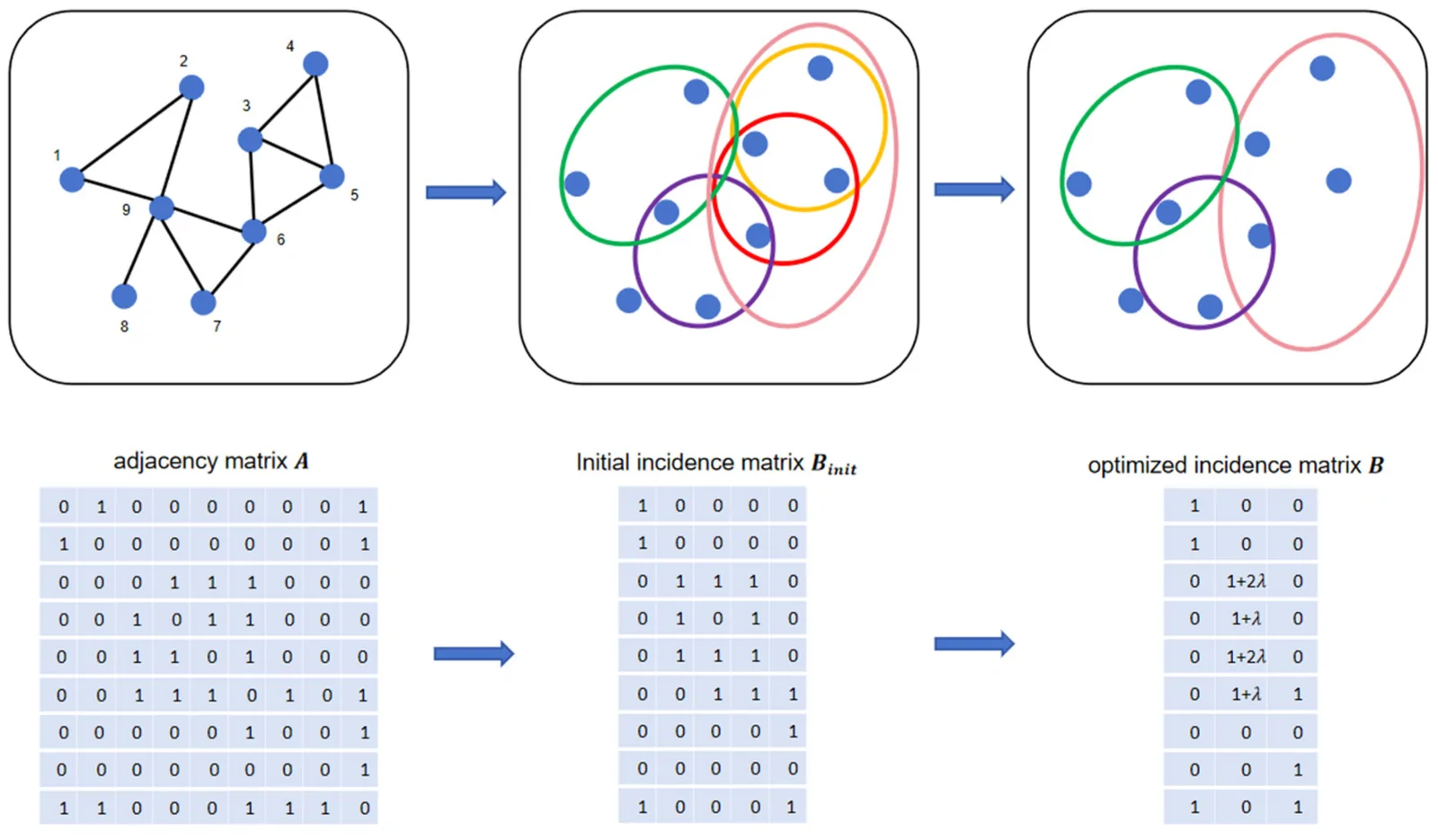
Illustration of the hypergraph construction and refinement process via nested motif pruning and node weight compensation.

To summarize the entire process described above, Figure 3 illustrates the complete workflow of our hypergraph construction and refinement. Algorithm 1 summarizes the detailed procedure of nested motif pruning and weight compensation.
**Algorithm 1:** Nested Motif Pruning and Node Weight Compensation**Input:** $\mathrm{Network}\;\mathrm{snapshot}\;G^{\left(t\right)}=(V,E^{\left(t\right)})$, compensation coefficient λ**Output:** $\mathrm{Refined}\;\mathrm{weighted}\;\mathrm{incidence}\;\mathrm{matrix}\;B^{\left(t\right)}$1: Extract 3-order closed motifs $M_{3}^{\left(t\right)}$ and 4-order closed motifs $M_{4}^{\left(t\right)}$ from $G^{\left(t\right)}$2: Initialize $M_{3,drop}^{\left(t\right)}$←Ø, and $M_{3,keep}^{\left(t\right)}$ ← $M_{3}^{\left(t\right)}$3: Build a Hash set $H_{3}$ to store vertex sets of all motifs in $M_{3}^{\left(t\right)}$ for O(1) lookup4: **// Step 1: Nested Motif Pruning**5: **for** each 4-order motif $\phi \in M_{4}^{\left(t\right)}$ **do**6:     Generate the combinations of 3-node subsets S={s⊂V(ϕ)∣|s|=3}7:     **for** each subset s∈S **do**8:         If $s\in H_{3}$ then9:             $M_{3,drop}^{\left(t\right)}\leftarrow M_{3,drop}^{\left(t\right)}\cup \{s\}$10:            $M_{3,keep}^{\left(t\right)}\leftarrow M_{3,keep}^{\left(t\right)}\setminus \{s\}$11:        End if12:    End for13:End for14:Retained motif set $M^{*(t)}\leftarrow M_{4}^{\left(t\right)}\cup M_{3,keep}^{\left(t\right)}$15:Map each motif in $M^{*(t)}$ to a hyperedge to form $E^{\left(t\right)}$16:Initialize incidence matrix $B^{\left(t\right)}$ of size $\left|V|\times |E^{\left(t\right)}\right|$ with zeros17:**// Step 2: Node Weight Compensation**18:for each hyperedge $\varepsilon_{k}^{\left(t\right)}\in E^{\left(t\right)}$ do19:    for each node $v_{i}\in \varepsilon_{k}^{\left(t\right)}$ do20:        Count $c^{\left(t\right)}(i,k)=|\{\tau \in M_{3,drop}^{\left(t\right)}|v_{i}\in V(\tau ),V(\tau )\subset \varepsilon_{k}^{\left(t\right)}\}|$21:        $B^{\left(t\right)}(i,k)=1+\lambda \cdot c^{\left(t\right)}(i,k)$22:    End for23:End for24:Return $B^{\left(t\right)}$

### 3.3. Dual-Channel Encoding

#### 3.3.1. GCN Channel

For the original network snapshot $G^{\left(t\right)}$ at time step t, this channel takes the adjacency matrix $A^{\left(t\right)}$ as input and adopts a GCN to encode low-order topological information of nodes. We first inject self-loops into the adjacency matrix, yielding ${\tilde{A}}^{\left(t\right)}=A^{\left(t\right)}+I_{N}$, where $I_{N}\in R^{N\times N}$ denotes the identity matrix and ${\tilde{D}}^{\left(t\right)}$ is the corresponding diagonal degree matrix of ${\tilde{A}}^{\left(t\right)}$. The symmetrically normalized adjacency matrix is thus computed as $({\tilde{D}}^{\left(t\right)}{)}^{-\frac{1}{2}}{\tilde{A}}^{\left(t\right)}({\tilde{D}}^{\left(t\right)}{)}^{-\frac{1}{2}}$. The forward propagation of the l-th GCN layer is formulated as:

(13) $$X_{g}^{\left(t,l+1\right)}=\sigma \left(({\tilde{D}}^{\left(t\right)}{)}^{-\frac{1}{2}}{\tilde{A}}^{\left(t\right)}({\tilde{D}}^{\left(t\right)}{)}^{-\frac{1}{2}}X_{g}^{\left(t,l\right)}W_{g}^{\left(l\right)}\right)$$

After L graph convolution layers, we obtain the low-order spatial feature representation at the current time step t: $S_{g}^{\left(t\right)}=X_{g}^{\left(t,L\right)}$.

To model the temporal dynamics of low-order structural features, $S_{g}^{\left(t\right)}$ is fed into a dedicated GRU unit in this channel to update the temporal hidden state:

(14) $$Z_{g}^{\left(t\right)}=GRU_{g}\left(S_{g}^{\left(t\right)},Z_{g}^{\left(t-1\right)}\right)$$

#### 3.3.2. HGCN Channel

For the refined hypergraph $H^{\left(t\right)}$, this channel utilizes an HGCN [22] to encode high-order structural information. It is worth noting that the node weight compensation coefficient introduced in Section 3.2.3 alters the numerical distribution of the incidence matrix. To prevent weighted values from distorting the intrinsic topological degree distribution of the network, we adopt a decoupled computation scheme that separates structural normalization from feature propagation in the HGCN.

We first derive a binarized incidence matrix $B_{bin}^{\left(t\right)}$ from the weighted incidence matrix $B^{\left(t\right)}$, with entries defined as:

(15) $$B_{bin}^{\left(t\right)}(i,k)=\left\{\begin{array}{cc} 1, & if\;B^{\left(t\right)}(i,k)>0,\\ 0, & otherwise. \end{array}\right.$$

With $B_{bin}^{\left(t\right)}$, we compute the unweighted vertex degree matrix $D_{v}^{\left(t\right)}$ and the hyperedge degree matrix $D_{e}^{\left(t\right)}$, which retain the original topological degree semantics. For representational simplicity, the hypergraph propagation matrix $P^{\left(t\right)}$ is defined as:

(16) $$P^{\left(t\right)}=(D_{v}^{\left(t\right)}{)}^{-\frac{1}{2}}B^{\left(t\right)}W(D_{e}^{\left(t\right)}{)}^{-1}(B^{\left(t\right)}{)}^{⊤}(D_{v}^{\left(t\right)}{)}^{-\frac{1}{2}}$$

where W denotes hyperedge weight matrix. The forward propagation of the l-th HGCN layer is formulated as:

(17) $$X_{h}^{\left(t,l+1\right)}=\sigma \left(P^{\left(t\right)}X_{h}^{\left(t,l\right)}W_{h}^{\left(l\right)}\right)$$

After L layers of hypergraph convolution, we obtain the high-order spatial feature representation at time step t: $S_{h}^{\left(t\right)}=X_{h}^{\left(t,L\right)}$.

To model the temporal dynamics of high-order structural features, $S_{h}^{\left(t\right)}$ is further fed into an independent GRU unit to update the temporal hidden state:

(18) $$Z_{h}^{\left(t\right)}=GRU_{h}\left(S_{h}^{\left(t\right)},Z_{h}^{\left(t-1\right)}\right)$$

By equipping ${GRU}_{g}$ and ${GRU}_{h}$ with independent parameter spaces, we fully isolate the propagation paths of low-order and high-order information, which avoids mutual interference during temporal evolution modeling.

### 3.4. Adaptive Fusion

After completing the encoding at time step t, we obtain node representations from two dimensions: the low-order structural channel representation $Z_{g}^{\left(t\right)}$, which focuses on characterizing the patterns of low-order binary pairwise interactions, and the high-order structural channel representation $Z_{h}^{\left(t\right)}$, which emphasizes the evolutionary patterns of high-order structures. To adaptively integrate the two types of information, we design a learnable gating fusion mechanism:

(19) $$\Gamma^{\left(t\right)}=\sigma \left(W_{f}[Z_{g}^{\left(t\right)}∥Z_{h}^{\left(t\right)}]+b_{f}\right)$$

where ∥ denotes feature concatenation, $W_{f}$ is the learnable gating weight matrix, $b_{f}$ is the bias vector, and $\Gamma^{\left(t\right)}\in R^{N\times d}$ is the generated adaptive gating matrix.

The final comprehensive node representation $Z^{\left(t\right)}$ is derived by weighting and fusing dual-channel features via the gating matrix:

(20) $$Z^{\left(t\right)}=\Gamma^{\left(t\right)}⊙Z_{g}^{\left(t\right)}+(1-\Gamma^{\left(t\right)})⊙Z_{h}^{\left(t\right)}$$

where ⊙ denotes the Hadamard product, and 1 is an all-ones matrix with dimensions matching those of $\Gamma^{\left(t\right)}$.

### 3.5. Link Prediction

We cast the link prediction task as a binary classification problem. For each candidate node pair $\left(v_{i},v_{j}\right)$, we first retrieve their corresponding node embeddings $z_{i}^{\left(t\right)}$ and $z_{j}^{\left(t\right)}$, and construct a symmetric edge representation $p_{ij}^{\left(t\right)}$ as follows:

(21) $$p_{ij}^{\left(t\right)}=\left[\left|z_{i}^{\left(t\right)}-z_{j}^{\left(t\right)}|∥(z_{i}^{\left(t\right)}⊙z_{j}^{\left(t\right)}\right)\right]$$

The existence probability of the edge is then calculated by an MLP [23], yielding the predicted probability ${\hat{y}}_{ij}^{\left(t+1\right)}=\sigma \left(MLP(p_{ij}^{\left(t\right)})\right)$.

For model training, we adopt the standard Binary Cross-Entropy (BCE) loss to perform end-to-end optimization over all training samples:

(22) $$L=-\frac{1}{\left|E_{train}\right|}\sum_{(i,j)\in E_{train}}\left(y_{ij}^{\left(t+1\right)}\log{\hat{y}}_{ij}^{\left(t+1\right)}+(1-y_{ij}^{\left(t+1\right)})\log(1-{\hat{y}}_{ij}^{\left(t+1\right)})\right)$$

where $E_{train}$ is the set of positive and negative sample pairs in the training set; $y_{ij}^{\left(t+1\right)}$ is the ground-truth topological label, such that $y_{ij}^{\left(t+1\right)}=1$ if an actual edge exists between node $v_{i}$ and node $v_{j}$ at time step t+1, and 0 otherwise.

## 4. Experiments and Analysis

### 4.1. Datasets

To evaluate the performance of the DC-GHCN model, five publicly available dynamic network datasets are selected to conduct the experiments. The detailed information for each dataset is summarized in Table 1.

Fb-Forum [24]: A network dataset originating from a Facebook forum. Nodes represent users, and edges denote their interactions.

Bitcoin-OTC [25]: A trust network constructed from a Bitcoin over-the-counter (OTC) trading platform. Nodes represent anonymous users participating in Bitcoin transactions, and edges denote the transactions or trust-rating behaviors initiated between users.

UCI [26]: This dataset records the private message-passing history among students within an online social network at the University of California, Irvine (UC Irvine).

Email-Eu [27]: An email communication network within a European research institution. Nodes represent institution members, and edges indicate email exchanges among them.

M-Overflow [27]: This dataset logs the temporal interaction behaviors among users on the MathOverflow Q&A platform. Nodes represent registered users, and edges denote interactions between them (e.g., answering questions, commenting, or mentioning).

**Figure 4:**
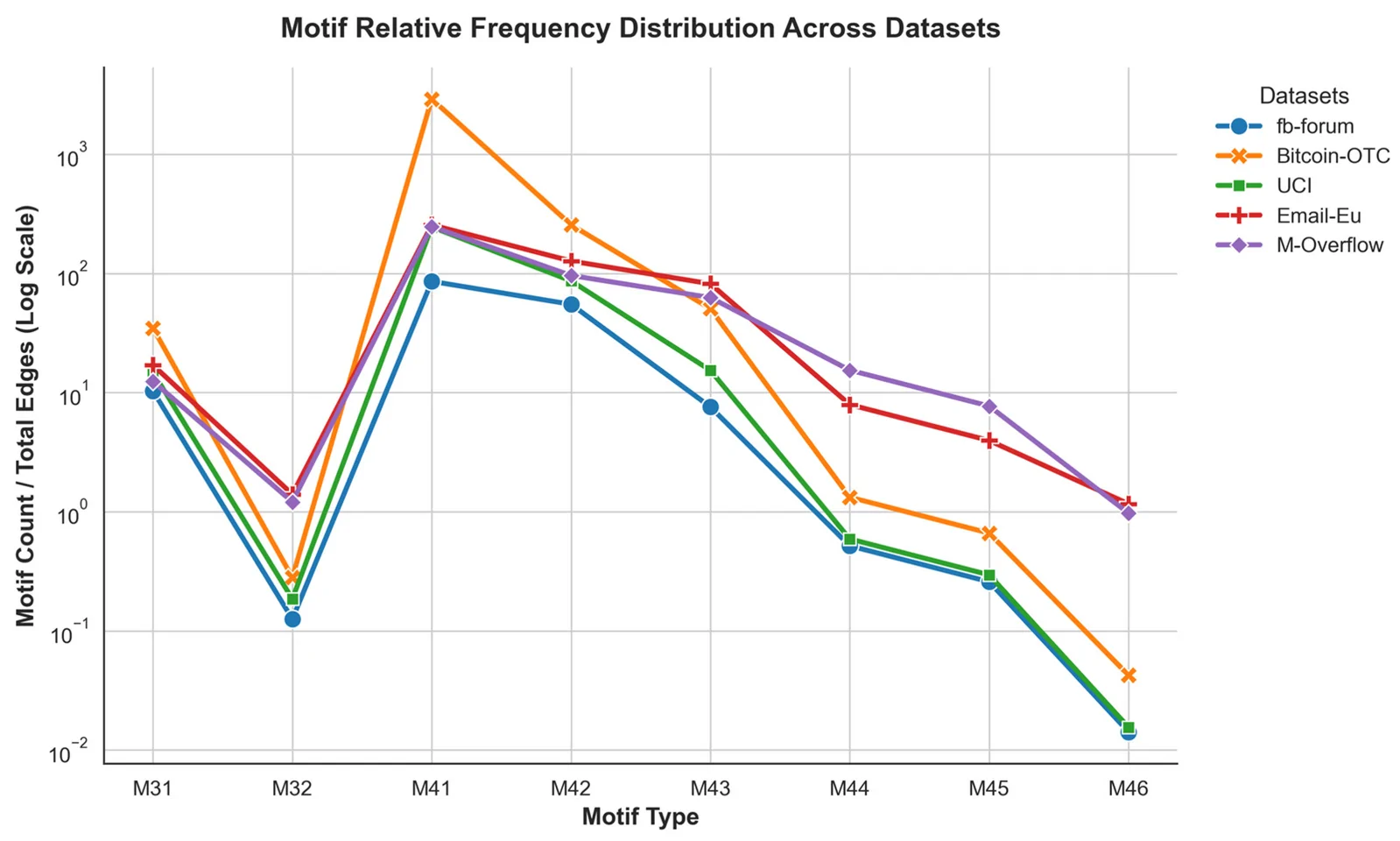
The quantity ratio of each motif type to the total number of edges across five datasets.

Additionally, we computed the quantity ratio of each motif type to the total number of edges across all datasets, with the results illustrated in Figure 4. As can be observed, non-closed motifs (M31, M41, M42, M43) far outnumber edges in all datasets. If all motif types were adopted for hyperedge mapping, the hypergraph scale would grow dramatically, incurring prohibitive computational overhead. For this reason, we discard non-closed motifs and construct the hypergraph exclusively with closed motifs, which guarantees both the representational effectiveness and computational efficiency of high-order feature extraction.

### 4.2. Baseline Models

We selected the following eight baseline models for comparison with the proposed DC-GHCN model:

AA [7]: A classic heuristic baseline that estimates the probability of edge formation by quantifying the importance of shared neighbors between two nodes.

GAT [10]: A representative static graph neural network that aggregates neighborhood information via attention mechanisms to learn latent node embeddings.

GC-LSTM [13]: A classical dynamic graph model that integrates graph convolution into the LSTM architecture to jointly capture network structural features and temporal evolutionary patterns.

EvolveGCN-O [14]: A variant of EvolveGCN. Instead of directly evolving node embeddings, it leverages recurrent neural networks to update GCN parameter matrices along the temporal dimension.

EvolveGCN-H [14]: The other variant of EvolveGCN. Distinct from the -O version, it treats GCN parameter matrices as the hidden states of recurrent units and updates parameters jointly with node representations.

DySAT [15]: A self-attention-based dynamic graph representation learning model. It alternately stacks structural and temporal self-attention layers to learn intra-snapshot structural relations and cross-snapshot evolution patterns simultaneously.

TMAGCN [16]: A motif-aware dynamic graph model that explicitly incorporates high-order local topological structures into node representations for joint spatio-temporal modeling.

MaskDGNN [28]: A state-of-the-art dynamic graph neural network based on self-supervised masked learning. It captures latent dependencies of nodes and edges in both spatial and temporal dimensions via a masked reconstruction mechanism.

### 4.3. Experimental Setup and Evaluation Metrics

#### 4.3.1. Experimental Setup

To preserve the natural temporal order and avoid future information leakage, we split the full snapshot sequence into training, validation, and test sets in strict chronological order, with a proportional split of 70%, 15%, and 15%, respectively. For each prediction step, edges in the target snapshot are treated as positive samples, while negative samples are randomly drawn at a 1:1 ratio from node pairs that are not connected in the corresponding target snapshot, thereby preventing target positive edges from being mislabeled. Negative samples are generated independently for each snapshot and each experimental run. Within each run, the validation and test negative sets remain fixed and are shared by all compared models. Experiments are repeated five times with different random seeds, and the results are reported as the mean ± standard deviation. The key hyperparameter configurations of our model are summarized in Table 2.

#### 4.3.2. Evaluation Metrics

To comprehensively evaluate the performance of each model on the dynamic link prediction task, we adopt AUC (Area Under the Receiver Operating Characteristic Curve) and AP (Average Precision) as evaluation metrics. Both metrics range from 0 to 1, with higher values corresponding to superior prediction performance.

AUC characterizes the overall ranking and discriminative power of the model between positive and negative samples. Probabilistically, it measures the probability that a randomly sampled ground-truth edge (positive sample) receives a higher predicted score than a randomly sampled non-edge (negative sample). For discrete computation in link prediction, each pairwise comparison yields a score of 1 if the positive sample outscores the negative sample, and 0.5 for tied scores. The formal definition is:

(23) $$AUC=\frac{n_{1}+0.5n_{2}}{n}$$

where n denotes the total number of independent comparisons (equal to the product of the number of positive samples and the number of negative samples in the test set); $n_{1}$ represents the number of times the predicted score of the positive sample is strictly greater than that of the negative sample; and $n_{2}$ indicates the number of times the predicted score of the positive sample is equal to that of the negative sample.

AP comprehensively considers the relationship between Precision and Recall, exhibiting higher sensitivity in dynamic network link prediction scenarios where positive and negative samples are highly imbalanced. Its calculation formula is as follows:

(24) $$AP=\frac{1}{N_{pos}}\sum_{k=1}^{N}P(k)\times \Delta r(k)$$

where N is the total number of samples in the test set (including both positive and negative samples); $N_{pos}$ is the total number of actual edges (positive samples) in the test set; k represents the cutoff rank after sorting the samples in descending order based on their predicted scores; P(k) denotes the precision among the top k samples; and Δr(k) is an indicator function, where Δr(k)=1 if the sample ranked k-th is an actual edge, and Δr(k)=0 otherwise.

### 4.4. Experimental Results and Analysis

This section compares the performance of the proposed DC-GHCN model with baseline models on the Fb-Forum, Bitcoin-OTC, UCI, Email-Eu, and M-Overflow datasets. The detailed experimental results in terms of AUC and AP are presented in Table 3 and Table 4, respectively.

The proposed DC-GHCN outperforms all baseline methods on every dataset. Traditional heuristic model AA achieves the weakest overall performance, confirming that local neighborhood similarity alone is insufficient to capture the intricate temporal evolutionary patterns of dynamic graphs. The static graph neural network GAT yields limited performance, as it is inherently constrained by the lack of an explicit temporal modeling mechanism. While mainstream dynamic graph models such as GC-LSTM, EvolveGCN, DySAT, and MaskDGNN are capable of modeling structural temporal evolution, their representation learning is built on standard pairwise topology, resulting in a notable performance gap relative to DC-GHCN. This observation demonstrates that high-order structures in real-world dynamic networks encode critical evolutionary information that cannot be substituted by low-order pairwise edges. TMAGCN further incorporates high-order topological information, yet it conducts joint spatio-temporal modeling without decoupling low-order and high-order components, thus neglecting the divergent evolutionary rates and patterns between the two types of structures. In contrast, the consistent performance superiority of DC-GHCN empirically validates the effectiveness of the proposed dual-channel parallel encoding architecture.

### 4.5. Parameter Sensitivity Analysis

**Figure 5:**
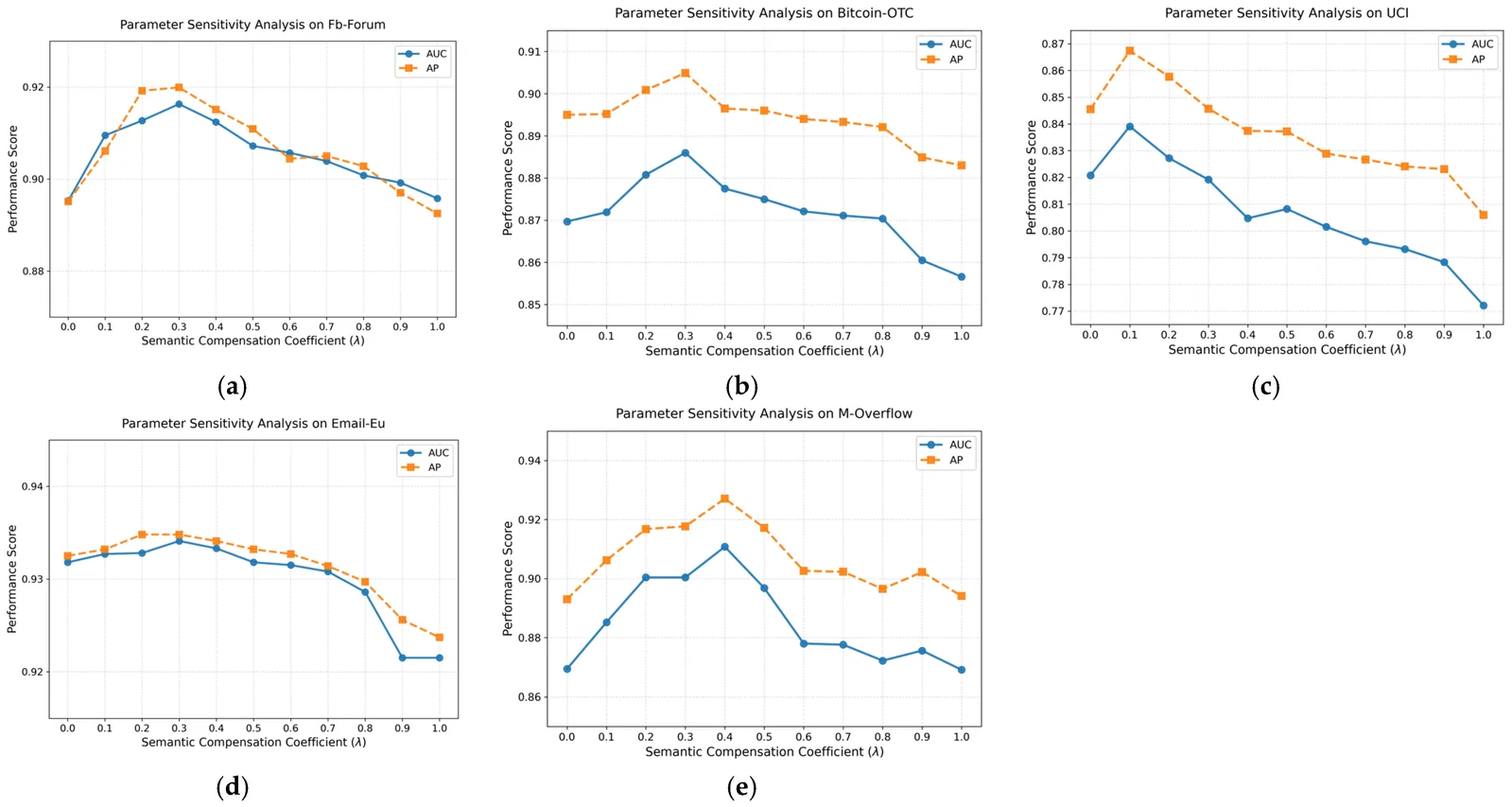
Sensitivity analysis of the semantic compensation coefficient λ for link prediction performance on different datasets: (**a**) Fb-Forum; (**b**) Bitcoin-OTC; (**c**) UCI; (**d**) Email-Eu; (**e**) M-Overflow.

In DC-GHCN, the compensation coefficient λ governs the extent to which topological information from pruned nested motifs is incorporated into the node incidence weights. To explore its influence on prediction performance in depth, we perform a sensitivity analysis of λ, with the results plotted in Figure 5.

As observed, when λ=0, corresponding to the setting with only nested motif pruning and no weight compensation, the model yields the lowest performance across all datasets. This suggests that while nested motif removal reduces structural redundancy at the global level, it inevitably diminishes local dense interaction information at the node level—notably, the effective connection strength of nodes is weakened by the pruning process. As λ increases, performance consistently improves across all five datasets, and peaks within the range λ∈[0.1,0.4]. This result confirms that compensating for pruned information via node weights is effective in recovering lost local structural signals. Nevertheless, performance declines consistently once λ exceeds the optimal threshold, forming a distinct inverted U-shaped curve in the sensitivity analysis. The underlying reason is that an overly large compensation coefficient disrupts the structural normalization of the hypergraph incidence matrix. During message passing, this causes the model to concentrate its receptive field excessively on highly overlapping local dense subgraphs, thereby overshadowing the general topological features of non-nested regions. Overall, an appropriately set compensation weight strikes the optimal trade-off between removing topological redundancy and retaining local structural information.

### 4.6. Ablation Experiment

**Figure 6:**
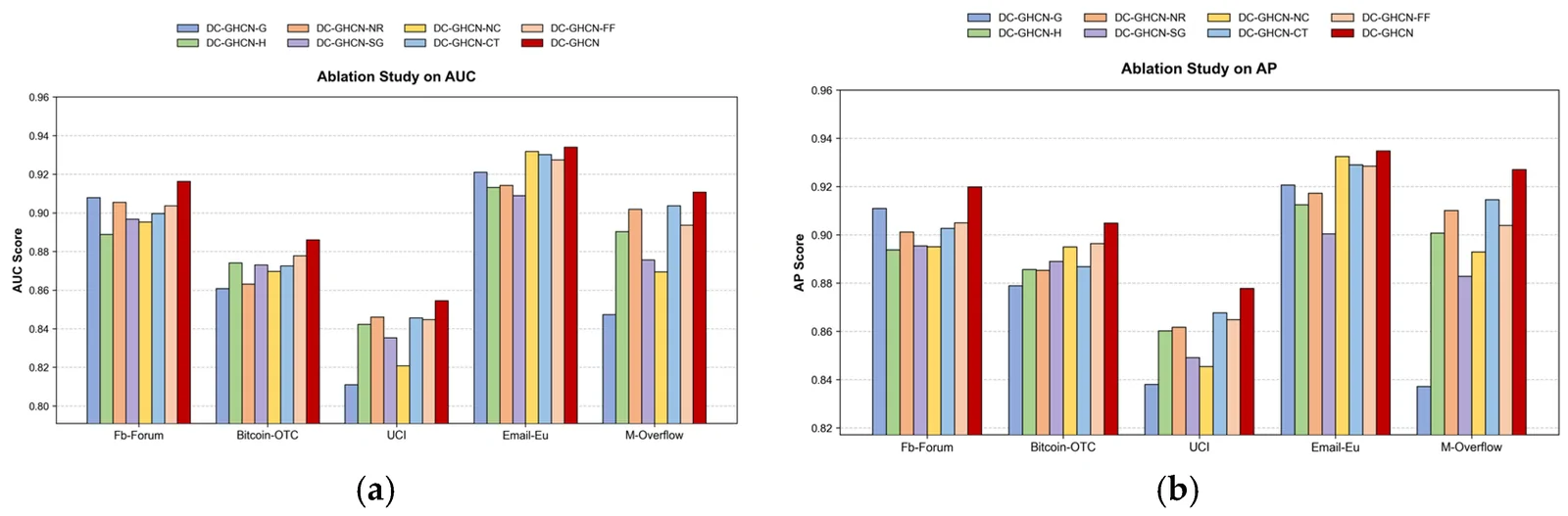
Ablation results of DC-GHCN and its variants across five datasets: (**a**) AUC results; (**b**) AP results.

To verify the effectiveness of the core design components within the DC-GHCN model, we conducted an ablation study, with the results presented in Figure 6. The specific ablation variants are designed as follows:

DC-GHCN-G: Low-order only variant, with the HGCN channel removed.

DC-GHCN-H: High-order only variant, with the GCN channel removed.

DC-GHCN-NR: Full model without nested motif pruning, where all extracted closed motifs are directly used for hypergraph construction.

DC-GHCN-SG: Dual-channel variant with shared GRU parameters, where both branches update temporal states with the same set of parameters.

DC-GHCN-NC: Full model with nested motif pruning but without node-weight compensation.

DC-GHCN-CT: Dual-channel variant with the adaptive gating mechanism replaced by direct concatenation, followed by a linear projection to maintain the original representation dimension.

DC-GHCN-FF: Dual-channel variant with the adaptive gating mechanism replaced by fixed equal-weight fusion, where both channels are assigned a weight of 0.5.

Figure 6a,b present the AUC and AP results, respectively, of DC-GHCN and its ablation variants across five real-world dynamic network datasets. Overall, the two metrics exhibit similar performance trends. First, the full DC-GHCN consistently outperforms the two single-channel variants, DC-GHCN-G and DC-GHCN-H, indicating that low-order and high-order structural information provide complementary contributions to dynamic link prediction. Second, DC-GHCN achieves better performance than DC-GHCN-NR, suggesting that directly retaining all extracted closed motifs may introduce redundant propagation paths, whereas nested motif pruning helps refine the high-order structural representation. Meanwhile, the performance improvement over DC-GHCN-NC demonstrates that node-weight compensation can alleviate the loss of useful local structural information caused by motif pruning. Third, the full model outperforms DC-GHCN-SG, supporting the use of independently parameterized GRUs to capture the distinct temporal evolution patterns of low-order and high-order structures. Finally, DC-GHCN also achieves better results than DC-GHCN-CT and DC-GHCN-FF. This indicates that direct concatenation and fixed equal-weight fusion cannot fully capture the varying contributions of the two channels, whereas the adaptive gating mechanism can dynamically balance low-order and high-order representations to produce more informative node embeddings.

### 4.7. Comparative Analysis of Hypergraph Construction Strategies

To further validate the rationality of the proposed hypergraph construction strategy, this section conducts comparative experiments against four heuristic strategies for hypergraph construction.

All_Motifs: Extracts all 3-order and 4-order motifs within the local neighborhoods as hyperedges.

Ego_Net [29]: Groups each central node in the network along with all its 1-order neighbors into a single hyperedge (restricting the total number of nodes within a hyperedge to be strictly greater than 3 to ensure its high-order nature).

Degree_E [30]: Partitions nodes possessing identical degree values in the network into the same hyperedge (excluding peripheral nodes with a degree of 1).

Simplex [31]: Exclusively extracts 3-order and 4-order simplices that satisfy the fully connected condition within the network as hyperedges.

Ours: Extracts closed motifs to construct hyperedges while strictly executing the nested motif pruning and node weight compensation strategies.

**Figure 7:**
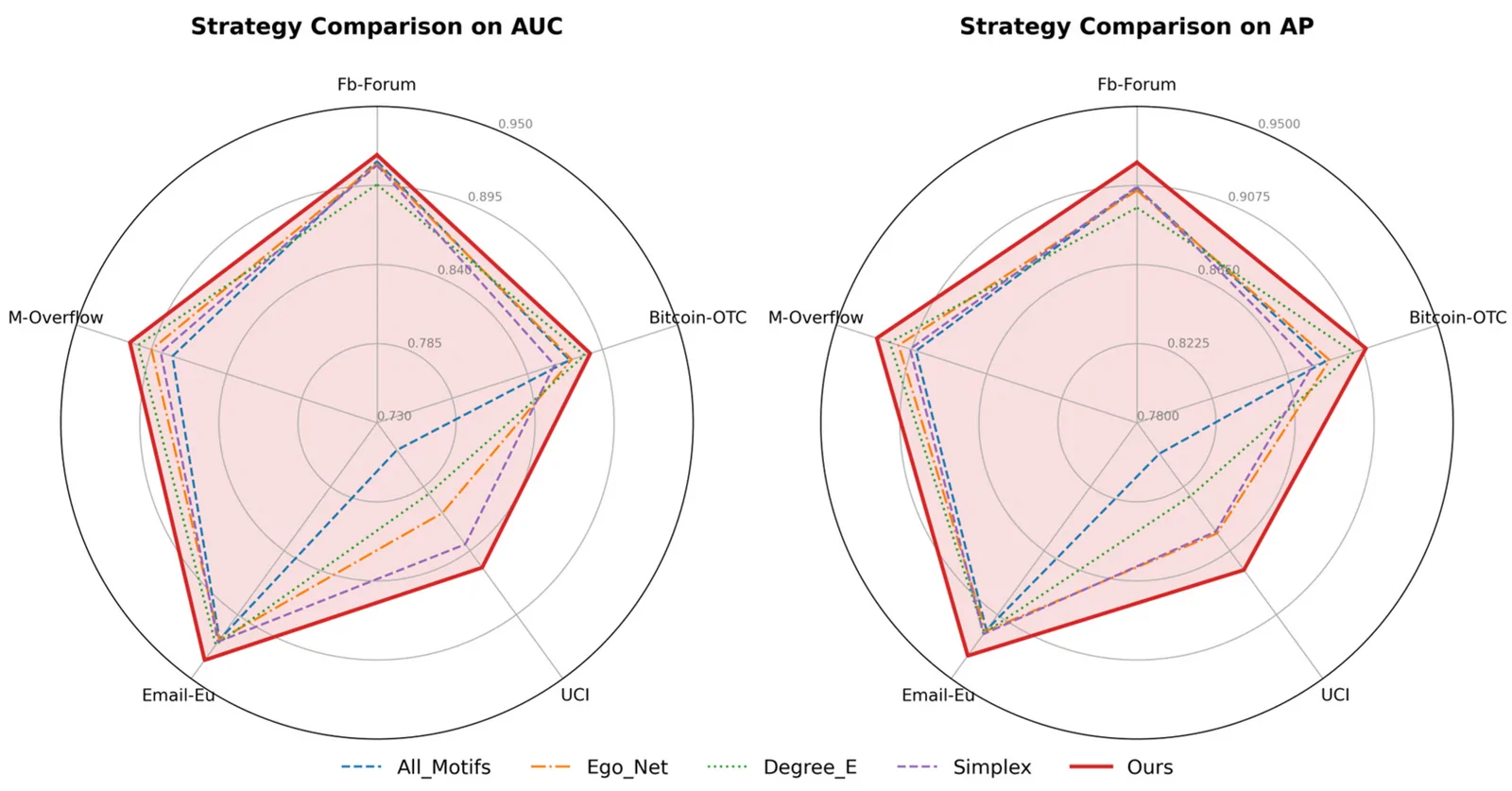
Prediction performance across different datasets utilizing various hypergraph construction strategies.

As shown in Figure 7, our proposed hypergraph construction strategy achieves the highest AUC and AP scores across all five datasets, outperforming all competing heuristic approaches.

The Degree_E strategy performs unstably. This is because this strategy solely relies on the statistical degree distribution, forcibly partitioning nodes with identical degrees into the same hyperedge, completely severing the network’s genuine local connectivity. In dynamic evolution, identical degrees do not imply the existence of actual physical interactions or similar evolutionary trajectories between nodes. Such “pseudo-high-order” relationships, detached from topological proximity, introduce severe topological noise during message passing. Although the Ego-Net strategy can capture the first-order local structure of central nodes, the massive overlap among Ego-Nets in complex networks means that directly mapping them into hyperedges leads to severe information redundancy. During the aggregation process of Hypergraph Convolution (HGCN), this indiscriminate, expansive neighborhood aggregation is highly prone to triggering the over-smoothing problem, thereby masking the genuine evolutionary features of high-order groups. The Simplex and All_Motifs strategies represent two extremes. The construction conditions of Simplex are overly stringent (retaining exclusively fully connected simplices), causing the model to lose a vast amount of locally connected patterns with actual physical significance in the network, such as cyclic structures. Conversely, while the All_Motifs strategy captures comprehensive high-order patterns, it introduces numerous sparse and uninformative subgraphs and fails to address the inherent topological nesting phenomenon among motifs of different orders, inevitably bringing redundancy to the feature propagation paths.

Overall, our proposed strategy strikes a favorable balance: closed motif extraction identifies cohesive, meaningful high-order interaction groups, while nested motif pruning and node weight compensation further reduce redundant propagation and preserve local structural information, ultimately producing high-quality hypergraphs well suited for dynamic link prediction.

### 4.8. Time Complexity Analysis

To comprehensively evaluate the computational efficiency of various models on the dynamic link prediction task, this section conducts a time complexity analysis for all compared algorithms, with the analytical results summarized in Table 5.

Suppose the dynamic network consists of T discrete snapshots, with each snapshot containing an average of N nodes and E edges. Let v denote the average node degree, d be the hidden layer dimensionality of the neural network, and $\left|E_{test}\right|$ represent the number of target edges for prediction. For models that accumulate historical snapshots into a static graph input, let $E_{acc}$ denote the final total number of edges in the accumulated graph. The theoretical time complexity analysis for all compared models is presented in Table 5.

The computational overhead of the proposed model is divided into two components: offline pre-computation and online training. To explicitly detail the exact computational cost of each preprocessing step, the offline phase consists of: (1) motif extraction($O(|E|\cdot v^{2})$), where v denotes the average node degree; (2) nested motif pruning via constant-time Hash lookups ($O(|M_{4}|)$); and (3) node weight compensation ($O(|M^{*}|\cdot |M_{3,drop}|)$). Therefore, the overall complexity of offline motif detection and nested motif pruning is approximately $O(|E|\cdot v^{2}+|M^{*}|\cdot |M_{3,drop}|)$; this process is executed exclusively once prior to training. During the online forward propagation phase, the complexities of graph convolutional aggregation for the dual channels are O(E⋅d) and O(M⋅d), respectively (where M represents the number of hyperedges after redundancy pruning). Incorporating the GRU temporal evolution and prediction scoring processes, the overall time complexity for online training is $O(T\cdot ((E+M)\cdot d+N\cdot d^{2})+|E_{test}|\cdot d^{2})$.

Overall, compared with all baselines, DC-GHCN has higher time complexity than static heuristic methods, but it offloads all time-intensive local motif detection and nested pruning operations to the offline preprocessing stage. For the core online training and inference phase, the spatio-temporal message passing complexity scales linearly with the number of optimized hyperedges M. This design effectively avoids the quadratic scaling of computational cost with the number of time steps.

## 5. Conclusions

Existing dynamic link prediction methods face two limitations: they often introduce severe information redundancy when incorporating motif information, and their coupled modeling paradigms lead to over-coupling of temporal features between low-order and high-order structures. To address these issues, we propose DC-GHCN, a novel dynamic link prediction model based on a dual-channel architecture of GCN and HGCN. The model delivers improved prediction performance through the collaboration of three core modules. The hypergraph construction module adopts nested motif pruning and node weight compensation strategies, which eliminate redundant information while preserving the connection strength of nodes. The dual-channel encoding module extracts low-order and high-order spatio-temporal features separately via GCN, HGCN, and GRU. Adaptive gating mechanism fuses the dual-channel node representations to generate final embeddings for link prediction. Experimental analyses on five real-world dynamic network datasets demonstrate that DC-GHCN effectively extracts and leverages both low-order and high-order structural information, delivering overall link prediction performance that outperforms mainstream baseline models.

Although the five datasets considered in this study cover diverse interaction scenarios, they primarily belong to the category of dynamic interaction networks. Since the proposed hypergraph construction relies on closed high-order motifs, its effectiveness may depend to some extent on the prevalence of such structures in the target network. For very large or densely connected graphs, motif extraction may also increase the offline preprocessing cost, although this procedure is performed only once before model training. Moreover, the current framework is mainly evaluated on homogeneous and unweighted dynamic graphs with relatively stable node sets, and its potential extension to directed, weighted, heterogeneous, or rapidly evolving networks deserves further exploration. Future work will further evaluate DC-GHCN on dynamic graphs from substantially different domains and investigate more flexible and efficient motif-extraction strategies, thereby providing broader empirical support for its generalizability and further improving its applicability and scalability.

## Figures and Tables

**Table 1:** Topological characteristics of the datasets. N denotes the total number of nodes in the network, E represents the total number of edges, and T indicates the number of time steps.

Dataset	N	E	T
Fb-Forum	899	33,720	23
Bitcoin-OTC	5881	35,592	15
UCI	1899	59,835	27
Email-Eu	986	332,334	26
M-Overflow	24,818	506,550	78

**Table 2:** Complete hyperparameter configuration.

Hyperparameter	Value	Description
$L_{g}$	2	Number of graph convolutional layers
$L_{h}$	2	Number of hypergraph convolutional layers
*η*	0.01	Adam optimizer learning rate
$W_{d}$	5 × 10^−4^	L2 regularization coefficient
$d_{1}$	64	First GCN/HGCN layer dimension
$d_{2}$	32	Second GCN/HGCN layer dimension
$E_{m}$	50	Maximum number of training epochs

**Table 3:** Comparison of AUC performance of various models on different datasets. Bold values indicate the best results.

Datasets	Fb-Forum	Bitcoin-OTC	UCI	Email-Eu	M-Overflow
AA	0.7181 ± 0.0000	0.8615 ± 0.0000	0.6510 ± 0.0000	0.9281 ± 0.0000	0.8239 ± 0.0000
GAT	0.8968 ± 0.0052	0.8653 ± 0.0041	0.7689 ± 0.0115	0.9017 ± 0.0048	0.8517 ± 0.0045
GC-LSTM	0.8693 ± 0.0085	0.8715 ± 0.0094	0.7581 ± 0.0162	0.8983 ± 0.0062	0.7394 ± 0.0198
EvolveGCN-O	0.8808 ± 0.0112	0.8209 ± 0.0086	0.7054 ± 0.0241	0.8417 ± 0.0084	0.7154 ± 0.0255
EvolveGCN-H	0.8787 ± 0.0094	0.8096 ± 0.0125	0.7527 ± 0.0286	0.8917 ± 0.0091	0.6942 ± 0.0224
TMAGCN	0.8901 ± 0.0031	0.8699 ± 0.0029	0.7580 ± 0.0045	0.9073 ± 0.0053	0.8284 ± 0.0042
DySAT	0.9049 ± 0.0028	0.8604 ± 0.0032	0.8296 ± 0.0084	0.9287 ± 0.0028	0.8616 ± 0.0048
MaskDGNN	0.9008 ± 0.0024	0.8740 ± 0.0045	0.8017 ± 0.0052	0.9295 ± 0.0037	0.8986 ± 0.0038
DC-GHCN	**0.9163** ± 0.0035	**0.8860** ± 0.0027	**0.8545** ± 0.0058	**0.9341** ± 0.0022	**0.9108** ± 0.0051

**Table 4:** Comparison of AP performance of various models on different datasets. Bold values indicate the best results.

Datasets	Fb-Forum	Bitcoin-OTC	UCI	Email-Eu	M-Overflow
AA	0.7301 ± 0.0000	0.8667 ± 0.0000	0.6540 ± 0.0000	0.9262 ± 0.0000	0.8327 ± 0.0000
GAT	0.8992 ± 0.0045	0.8918 ± 0.0082	0.8063 ± 0.0041	0.8923 ± 0.0041	0.8786 ± 0.0115
GC-LSTM	0.8744 ± 0.0092	0.8860 ± 0.0081	0.7969 ± 0.0165	0.9006 ± 0.0068	0.7919 ± 0.0215
EvolveGCN-O	0.8677 ± 0.0128	0.8236 ± 0.0105	0.7648 ± 0.0252	0.8319 ± 0.0079	0.7652 ± 0.0248
EvolveGCN-H	0.8658 ± 0.0105	0.8136 ± 0.0118	0.7969 ± 0.0224	0.8881 ± 0.0082	0.7252 ± 0.0295
TMAGCN	0.8932 ± 0.0038	0.8849 ± 0.0065	0.7909 ± 0.0048	0.9093 ± 0.0026	0.8518 ± 0.0046
DySAT	0.9075 ± 0.0034	0.8790 ± 0.0035	0.8512 ± 0.0098	0.9282 ± 0.0035	0.8894 ± 0.0052
MaskDGNN	0.9013 ± 0.0028	0.8863 ± 0.0031	0.8423 ± 0.0076	0.9301 ± 0.0032	0.9182 ± 0.0041
DC-GHCN	**0.9199** ± 0.0042	**0.9049** ± 0.0039	**0.8778** ± 0.0062	**0.9348** ± 0.0025	**0.9271** ± 0.0056

**Table 5:** Time complexity of the respective models.

Model	Time Complexity
AA	$O(|E_{test}|\cdot v)$
GAT	$O((E_{acc}\cdot d+N\cdot d^{2}))$
GC-LSTM	$O(T\cdot (E\cdot d+N\cdot d^{2}))$
EvolveGCN (-O/-H)	$O(T\cdot (E\cdot d+N\cdot d^{2}+d^{3}))$
TMAGCN	$O(T\cdot (E\cdot d+N\cdot d^{2})+T\cdot E\cdot v^{2})$
MaskDGNN	$O(T\cdot (E\cdot d+N\cdot d^{2})+T\cdot E_{mask}\cdot d)$
DySAT	$O(T\cdot (E\cdot d+N\cdot d^{2})+N\cdot T^{2}\cdot d)$
DC-GHCN	$O(T\cdot ((E+M)\cdot d+N\cdot d^{2})+|E_{test}|\cdot d^{2})$

## Data Availability

The data presented in this study are available upon request from the corresponding author.
